# Anthracycline-induced cardiomyopathy in childhood cancer survivors is associated with gene signatures of mitochondrial dysfunction—a COG ALTE03N1 report

**DOI:** 10.1186/s40959-025-00391-w

**Published:** 2025-10-06

**Authors:** Patrick J. Trainor, Purnima Singh, Xuexia Wang, Noha Sharafeldin, Liting Zhou, Lindsey Hageman, Saro H. Armenian, Jill P. Ginsberg, Douglas S. Hawkins, Frank G. Keller, Melissa M. Hudson, Joseph P. Neglia, Wendy Landier, Smita Bhatia

**Affiliations:** 1 Institute for Cancer Outcomes and Survivorship, University of Alabama at Birmingham, 1600 7th Ave S, Lowder 500, Birmingham, AL 35233 USA; 2 School of Medicine, Virginia Commonwealth University, Richmond, VA USA; 3 Department of Pediatrics, University of Alabama at Birmingham, Birmingham, AL USA; 4 Department of Biostatistics, Florida International University, Miami, FL USA; 5 Department of Population Sciences, City of Hope, Duarte, CA USA; 6 Department of Pediatrics, Children’s Hospital of Philadelphia, Philadelphia, PA USA; 7 Department of Pediatrics, Seattle Children’s, Seattle, WA USA; 8 Department of Pediatrics, Children’s Healthcare of Atlanta, Emory University, Atlanta, GA USA; 9 Department of Epidemiology and Cancer Control, St. Jude Children’s Research Hospital, Memphis, TN USA; 10 Department of Pediatrics, University of Minnesota, Minneapolis, MN USA

**Keywords:** Anthracyclines, Cardiomyopathy, Mitochondria, Childhood cancer, Survivorship

## Abstract

**Background:**

Anthracycline-induced cardiomyopathy is a leading cause of morbidity and mortality in survivors of childhood cancer. The mitochondrion is a key mediator of the cytotoxic effects of anthracycline treatment and mitochondrial dysfunction is a hallmark of cardiomyopathy and heart failure. We sought to evaluate whether mitochondrial processes differ between anthracycline-exposed childhood cancer survivors who developed cardiomyopathy versus those who did not.

**Methods:**

Peripheral blood was collected from 40 childhood cancer survivors who developed cardiomyopathy (cases) and 64 matched survivors who did not (controls). From these samples, gene expression was determined by RNA-Sequencing. Following bioinformatic processing, differential gene expression at the mRNA-level between cases and controls was determined. Human MitoCarta3.0, was utilized to determine if genes involved in mitochondrial processes were enriched for differential expression, and to identify differentially regulated mitochondrial pathways at the mRNA-level.

**Results:**

900 genes were identified as differentially expressed at the mRNA-level. The odds of a gene being differentially expressed were 2.43 times greater if it encodes for a protein that localizes to the mitochondria. Mitochondrial processes that were enriched for differentially expressed genes at the mRNA-level included electron transport chain complexes; reactive oxygen species metabolism; apoptosis, mitophagy, and autophagy; mitochondrial ribosome; mitochondrial transport and chaperones; and heme synthesis and processing. Additionally, we observed that a measure of pro-apoptotic balance (BAX to BCL-2 gene expression at the mRNA-level) was highest in severe cardiomyopathy, intermediate in mild cardiomyopathy, and lowest in survivors without cardiomyopathy.

**Conclusions:**

We observed substantial evidence that the expression of genes involved in mitochondrial processes differs in childhood cancer survivors who develop cardiomyopathy versus those who do not.

**Supplementary Information:**

The online version contains supplementary material available at 10.1186/s40959-025-00391-w.

## Background

Anthracyclines are a core component of treatment for multiple types of childhood cancer [1, 2]. Anthracycline-based therapy is used in 60% of children treated for cancer [1]. While anthracyclines have contributed to improved survival in several childhood cancers, anthracycline-induced cardiac toxicity increases the risk of cardiac dysfunction [3], with 5-year survival rates lower than 50% among those with anthracycline-induced cardiomyopathy [4]. The mechanisms of action of anthracyclines include increased mitochondrial iron accumulation [5, 6], and increased production of reactive oxygen species (ROS) [7]. Mitochondria play a critical role in mediating the cytotoxic effects of anthracyclines [8, 9]. Mitochondria have been recognized as the “powerhouse of the cell” for over a half century given the role of mitochondria in the synthesis of adenosine triphosphate (ATP) and localization of key catabolic and anabolic reactions within mitochondrion [10]. The generation of ROS that accompanies the citric acid cycle and the electron transport chain is an important physiological consequence of mitochondrial bioenergetics [11, 12]. While adaptive/homeostatic mitochondrial ROS-mediated signaling has been identified [12, 13], excessive mitochondrial ROS production can lead to mitochondrial and nuclear DNA damage [14], oxidative modification of lipids and proteins, and can trigger apoptosis (especially via the intrinsic apoptotic pathway) [15]. Direct effects of anthracyclines on the intrinsic (mitochondrial-mediated) apoptotic pathway via ROS generation have been noted [9] alongside the interaction between anthracyclines and iron that produces ROS [5, 6].

In addition to being a mediator of anthracycline cytotoxicity, mitochondrial dysfunction plays a role in *de novo* cardiomyopathy and heart failure [16, 17]. Decreasing fatty acid oxidation with increasing glycolysis is a hallmark of altered metabolism and mitochondrial bioenergetics in hypertrophied hearts [16, 18, 19]. Additionally, mitochondrial biogenesis, dynamics (fusion and fission), structure, and ion (especially calcium) regulation have been shown to be altered in heart failure [20].

Given the centrality of mitochondria in anthracycline-mediated cytotoxicity and mitochondrial dysfunction observed in *de novo* cardiac dysfunction and heart failure, we sought to evaluate whether mitochondrial processes differ between anthracycline-exposed childhood cancer survivors who did and did not develop cardiomyopathy. To do this, we utilized a matched case-control design in anthracycline-exposed childhood cancer survivors and measured gene expression at the mRNA-level from whole blood. After determining which genes were differentially expressed (DE) in survivors who developed anthracycline-induced cardiomyopathy, we sought to test two hypotheses: (1) whether there is an enrichment for genes coding for mitochondrially localized proteins among genes that are DE in anthracycline-induced cardiomyopathy, and (2) whether specific mitochondrial processes are enriched for genes that are DE in anthracycline-induced cardiomyopathy. This study represents a targeted secondary analysis of RNA-Seq data generated from the COG-ALTE03N1 cohort, previously analyzed in Singh, et al., where 36 differentially expressed genes were identified between survivors with and without anthracycline-induced cardiomyopathy [21]. That prior work highlighted pathways involving metabolism, inflammation, and structural remodeling. Building on those findings, the present study focuses specifically on genes encoding mitochondrially localized proteins and mitochondrial processes, given the established role of the mitochondrion in anthracycline toxicity.

## Methods

### Study design

Forty survivors who developed cardiomyopathy (cases) and 64 survivors who did not (matched controls) after anthracycline exposure were drawn from a Children’s Oncology Group study, COG-ALTE03N1 (NCT00082745). Cases were identified based on presentation with signs and symptoms of cardiomyopathy consistent with the AHA definition of cardiomyopathy [22]. In the absence of signs or symptoms, cases had echocardiographic features of left ventricular dysfunction (ejection fraction ≤ 40% and/or fractional shortening ≤ 28%). Lifetime anthracycline exposure was calculated by multiplying the cumulative dose (milligrams per square meter) of individual anthracyclines by a factor that reflects the drug’s cardiotoxic potential [23] and then summing the result. For each case, patients who had no signs or symptoms of cardiomyopathy after anthracycline exposure were randomly selected as controls with matching on primary cancer diagnosis, year of diagnosis (± 5 years), and race/ethnicity. The selected control subjects also needed to have a longer duration of cardiomyopathy-free follow-up compared with time from cancer diagnosis to cardiomyopathy for the corresponding case. To identify factors that differed between cases and controls, the distributions of age at primary cancer diagnosis, biological sex, anthracycline exposure, chest radiation, race/ethnicity, and primary cancer diagnosis were compared.

### Statistical methods

#### Identification of differentially expressed genes

Details of the RNA isolation, RNA sequencing, and bioinformatic processing have been described previously [21]. RNA was extracted from PAXgene Blood RNA tubes (Qiagen), and RNA integrity was confirmed using an Agilent Bioanalyzer. RNA libraries were prepared with the TruSeq RNA Sample Preparation Kit (Illumina) and sequenced on an Illumina NovaSeq 6000 platform with paired-end 100 bp reads. Reads were trimmed using TrimGalore, aligned to the GRCh38 reference genome using STAR, and quantified at the gene level using HTSeq-count. Following bioinformatic processing, *DESeq2* was utilized to determine evidence of differential expression (DE) between cases and controls [24]. Prior to fitting models using *DESeq2*, a pre-filter on counts removed genes with < 10 counts in >70% of all samples. In our previous work [21] this filter was applied within each group. The *DESeq2* models were adjusted for age at primary cancer diagnosis, biological sex, cumulative anthracycline dose (≥ 250 mg/m^2^ vs. < 250 mg/m^2^), and chest radiation. In this work, we considered q-values < 0.05 as significant evidence for differential expression which maintains the false discovery rate (FDR) at < 5%. A q-value of 0.05 implies that if all genes with a similar level of significance (q-value = 0.05) were declared to be differentially expressed, 5% of these assertions would be expected to be false positives.

#### Identification of mitochondrial processes enriched for differences

Differentially expressed genes (DEGs) coding for mitochondria-localized proteins were identified utilizing Human MitoCarta3.0 [25]. MitoCarta is a catalogue of proteins that have strong evidence of being localized to the mitochondria, leveraging experimental mass-spectrometry based localization, analysis of targeting signals, and literature review. We refer to DEGs that code for mitochondrially localized proteins as DEG-M and DEGs that do not code for mitochondria-localized proteins as DEG-NM. Mitochondrially localized proteins annotated as having “shared domains” in MitoCarta have domains that may also localize to other components of the cell were treated as DEG-M. To determine if there was evidence of dysregulated mitochondrial processes between cases and controls, we conducted two types of enrichment analyses. First, we evaluated whether there was an enrichment for DEGs between proteins localized to the mitochondria, versus not (i.e., DEG-M vs. DEG-NM). The second enrichment analysis evaluated whether there was enrichment for DEG-Ms in specific mitochondrial processes (referred to as pathways in MitoCarta). Given the hierarchical structure of the MitoCarta3.0 pathways/processes, we focused on terminal processes in our analyses of enrichment results with the following exceptions. Complexes of the oxidative phosphorylation system were not further sub-divided into subunits versus assembly factors, and protein import and sorting was not further sub-divided. Enrichment analyses were conducted using Gene Set Enrichment Analysis (GSEA) [26] using the magnitude of the case-control test statistic for ranking and implemented using the R package *fgsea* [27]. Statistical significance of mitochondrial process overrepresentation was determined by a one-sided permutation test procedure with *p*-values adjusted for preserving the FDR using the Benjamini-Hochberg method [28]. Genes that were in the leading edge of each GSEA running score were then identified as these genes are the core genes that provide evidence that for a mitochondrial process being enriched.

#### Secondary analysis comparing severe and mild cardiomyopathy

As a secondary analysis, we evaluated the pattern of differential expression between severe and mild cardiomyopathy. We fit adjusted models as described above with a three-level variable: control, mild cardiomyopathy, and severe cardiomyopathy. Severe cardiomyopathy was defined as symptomatic cardiac dysfunction with an ejection fraction ≤ 40% or fractional shortening ≤ 25%. 14 cases were classified as severe and 26 were classified as mild. We determined the fold-change differences in gene expression at the mRNA-level between severe and mild cardiomyopathy. To evaluate agreement between results of the secondary analysis comparing severe vs. mild cardiomyopathy to the primary analysis, the correlation between test statistics for the two analyses was then determined.

#### Secondary Analysis of BAX/BCL-2 Gene Expression Ratio at the mRNA-level

We evaluated the ratio of *BAX* to *BCL-2* gene expression at the mRNA-level as a marker of apoptotic susceptibility [29, 30]. After computing the ratio of the expression of these genes that code for mitochondrially localized proteins, the ratio was regressed separately on the case/control status and on the severity of cardiomyopathy. All secondary analyses conducted in the current study were considered exploratory in nature.

#### Data Availability

The data discussed in this article have been deposited in the NCBI Gene Expression Omnibus and are accessible through GEO Series accession number GSE218276 (https://www.ncbi.nih.gov/geo/query/acc.cgi?acc=GSE218276).

## Results

**Fig. 1:**
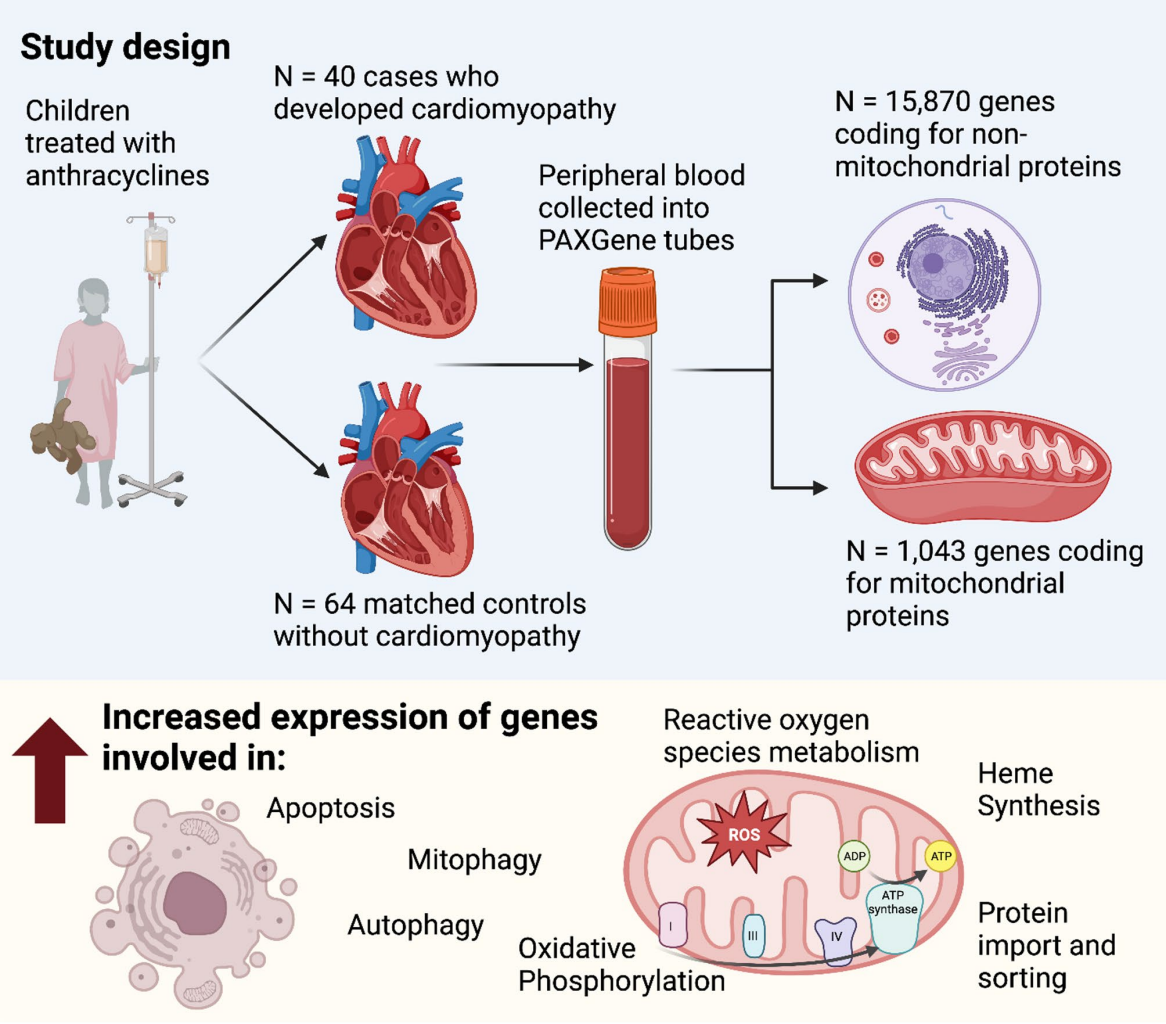
Cases with anthracycline-induced cardiomyopathy and matched controls were selected from a study of childhood cancer survivors treated with anthracyclines. Peripheral blood was collected into RNA-preserving PAXgene tubes. Poly-adenylated RNA was subjected to RNA-Sequencing. Gene expression at the mRNA-level was compared between cases and controls. Enrichment analyses were conducted for identifying mitochondrial processes enriched for differences in gene expression at the mRNA-level between cases and controls Figure created with BioRender

**Fig. 2:**
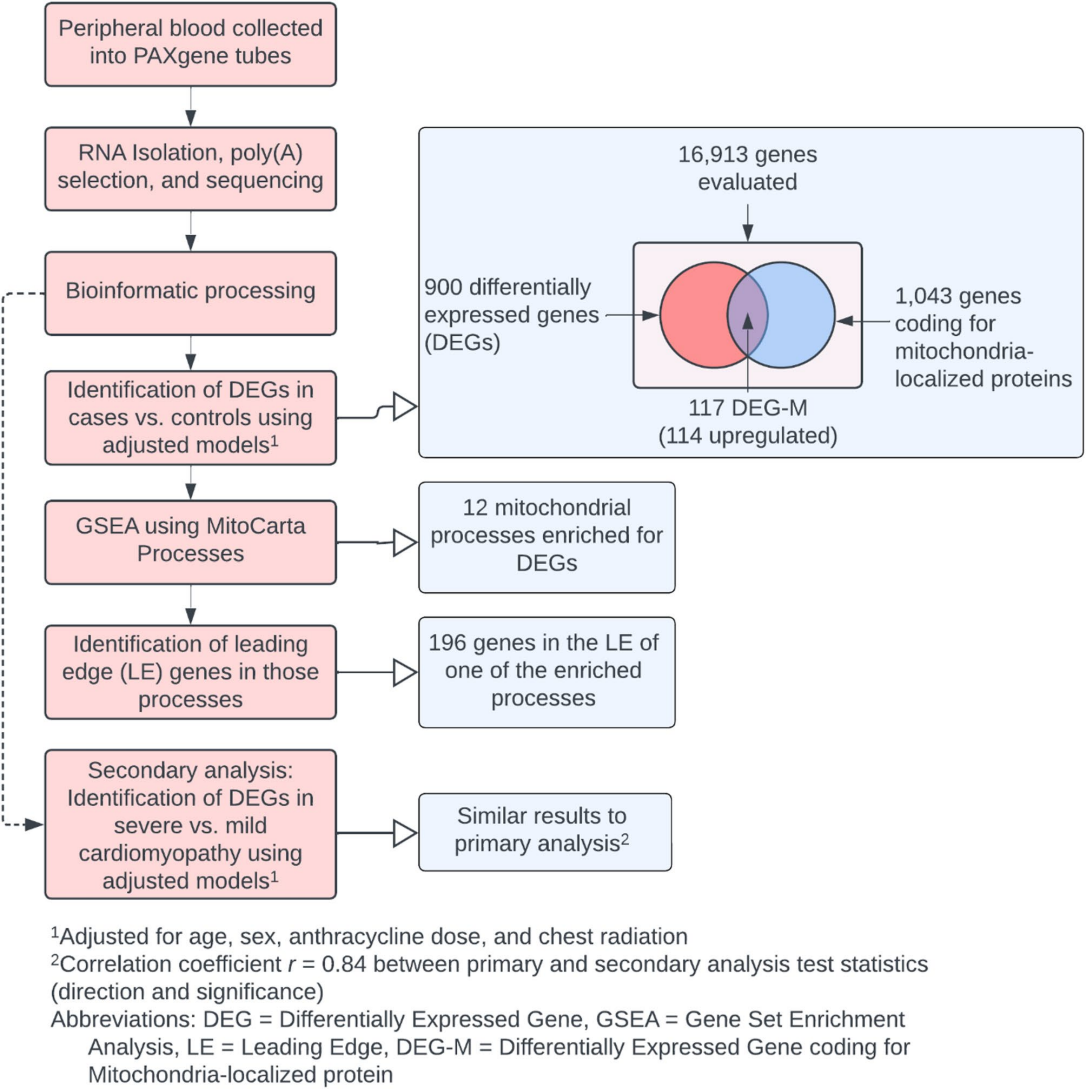
Analytical methodology (red color) employed in the current work and summary of results observed (blue)

A summary of the study design and important results is shown in the Fig. 1. A graphical summary of the analytical methodology employed in the current work is shown in Fig. 2.

### Participant characteristics

Characteristics of the study participants are shown in Table 1. Differences in the distributions of participant race/ethnicity and primary cancer diagnosis were not observed as these factors were matched between cases and controls. The median age at primary cancer diagnosis was 8.2 years in cases and 9.7 years in control (*p*-value = 0.97). At the time of blood sampling, the median age was 19.2 years in cases and 20.0 years in controls (*p*-value = 0.86). Cumulative anthracycline exposure > 250 mg/m^2^ was more prevalent in cases than controls (62.5% versus 35.9%, *p*-value = 0.009). Cases were more likely to have received chest radiation than controls (47.5% versus 20.3%, *p*-value = 0.005), however, the distribution of doses in those who received chest radiation was similar ([25th percentile, 75th percentile] = [1500, 4276] in cases versus [2100, 3960] in controls, *p*-value = 0.86).

**Table 1:** Characteristics of Anthracycline-exposed childhood cancer survivors. For continuous variables distributions were compared using the Wilcoxon rank-sum test. For categorical variables distributions were compared using fisher’s exact test

Variable		Case	Control	*p*-value
**Age at primary cancer diagnosis in years**, **median [25th**, **75th]**		8.2 [3.7, 13.7]	9.7 [3.4, 14.2]	0.97
**Age blood sampling in years**, **median [25th**, **75th]**		19.2 [15.9, 24.5]	20.0 [14.2, 25.8]	0.86
**Sex**, ***n*** **(%)**				
	Female	24 (60.0)	34 (53.1)	0.55
	Male	16 (40.0)	30 (46.9)	
**Cumulative Anthracycline exposure**, ***n*** **(%)**				
	< 250	15 (37.5)	41 (64.1)	0.009
	>=250	25 (62.5)	23 (35.9)	
**Chest Radiation**, ***n*** **(%)**				
	No	21 (52.5)	51 (79.7)	0.005
	Yes	19 (47.5)	13 (20.3)	
	Dose in cGy, median [25th, 75th]^a^	2550 [1500, 4276]	3000 [2100, 3960]	0.86
**Race / Ethnicity**, ***n*** **(%)**				
	Asian	3 (7.5)	3 (4.7)	0.96^b^
	Black	5 (12.5)	7 (10.9)	
	Hispanic	9 (22.5)	16 (25.0)	
	White	23 (57.5)	37 (57.8)	
	Mixed race / ethnicity	0 (0.0)	1 (1.6)	
**Primary diagnosis**, ***n*** **(%)**				
	Acute lymphoblastic leukemia	9 (22.5)	16 (25.0)	1.00^b^
	Acute myeloid leukemia	2 (5.0)	3 (4.7)	
	Ewing sarcoma	4 (10.0)	8 (12.5)	
	Hodgkin lymphoma	7 (17.5)	10 (15.6)	
	Kidney tumors	2 (5.0)	2 (3.1)	
	Neuroblastoma	5 (12.5)	8 (12.5)	
	Non-Hodgkin lymphoma	5 (12.5)	8 (12.5)	
	Osteosarcoma	4 (10.0)	7 (10.9)	
	Soft tissue sarcoma	2 (5.0)	2 (3.1)	
**Cardiovascular risk factor**, ***n*** **(%)**				
	No	24 (60.0)	62 (96.9)	< 0.0001
	Yes	14 (35.0)	2 (3.1)	
	Missing	2 (5.0)	0 (0.0)	
**Time from cancer diagnosis to study enrollment and blood sampling in years**, **median [25th**, **75th]**		11.1 [4.6, 18.2]	10.2 [7.4, 14.4]	0.80
**Time from cancer diagnosis to cardiac event for cases in years**, **median [25th**, **75th]**		5.4 [0.8, 12.2]	NA	

^a^In participants who were exposed to chest radiation

^b^Matching variable

### Differentially expressed genes and enriched mitochondrial processes

16,913 genes had sufficient expression for inclusion in our analysis and are summarized in Supplementary Table 1. Of these, 900 genes were differentially expressed between cases and controls (DEG). Of the 900 differentially expressed genes, 717 (79.7%) were upregulated in cases and 183 (20.3%) were downregulated. Of the 1,043 genes that coded for mitochondrial proteins, 117 were differentially expressed (DEG-M) with 114 (97.4%) of these being upregulated in cases. None of the 16 mitochondrial DNA-encoded genes had significant evidence of differential expression.

**Fig. 3:**
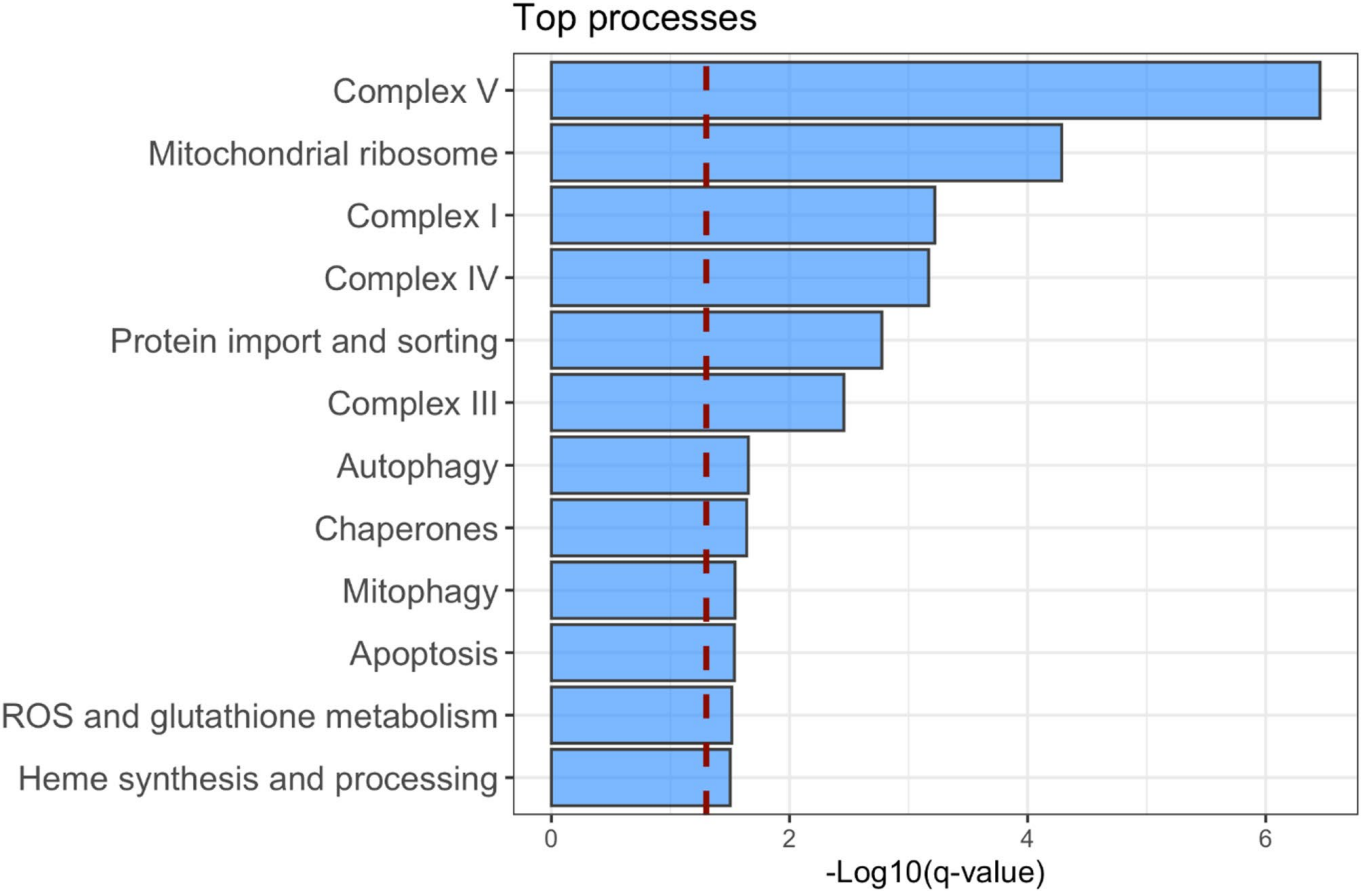
12 mitochondrial processes with significant evidence (q-value < 0.05) of enrichment for differentially expressed genes

Enrichment analyses were utilized to determine if there was evidence of dysregulated mitochondrial processes between cases and controls. In the evaluation of whether there was an enrichment for DEGs between proteins localized to the mitochondria, versus not (i.e., DEG-M vs. DEG-NM), the odds of DEG-M were 2.43 times greater when compared with DEG-NM (exact test *p*-value < 0.0001). There were 12 mitochondrial processes meeting our criteria for sufficient evidence of enrichment (q-value < 0.05) as shown in Fig. 3. The processes with the most significant evidence of enrichment for DEG-Ms included: Complex I, Complex III, Complex IV, Complex V; heme synthesis and processing; mitophagy, autophagy, and apoptosis; ROS and glutathione metabolism; mitochondrial ribosome; chaperone proteins; and protein import and sorting. Illustrative examples of the GSEA process for determining whether a mitochondrial process was enriched for DEGs are shown in Supplementary Fig. 1 highlighting the two processes with the greatest evidence for enrichment.

### Leading edge genes and direction of mitochondrial gene fold changes

We identified genes that were in the leading edge of the GSEA running score for mitochondrial processes with significant evidence of enrichment. These genes are the core genes that provide evidence that a mitochondrial process is enriched. 196 genes were in the leading edge of the running score for a significantly enriched mitochondrial process (Supplementary Table 2). Nearly 95% (186/196; 94.9%) of these genes exhibited greater average expression in cases versus control. Given this enrichment, we conducted an evaluation of the directionality of fold-changes for all genes included in the differential expression analysis (16,913 genes). As shown in Supplementary Fig. 2, while the average log-fold-change for genes not annotated as being involved in mitochondrial processes was 0.028 (95% CI, 0.024–0.031), the average log-fold-change for genes in mitochondrial processes was 0.117 (95% CI, 0.104–0.129).

**Fig. 4:**
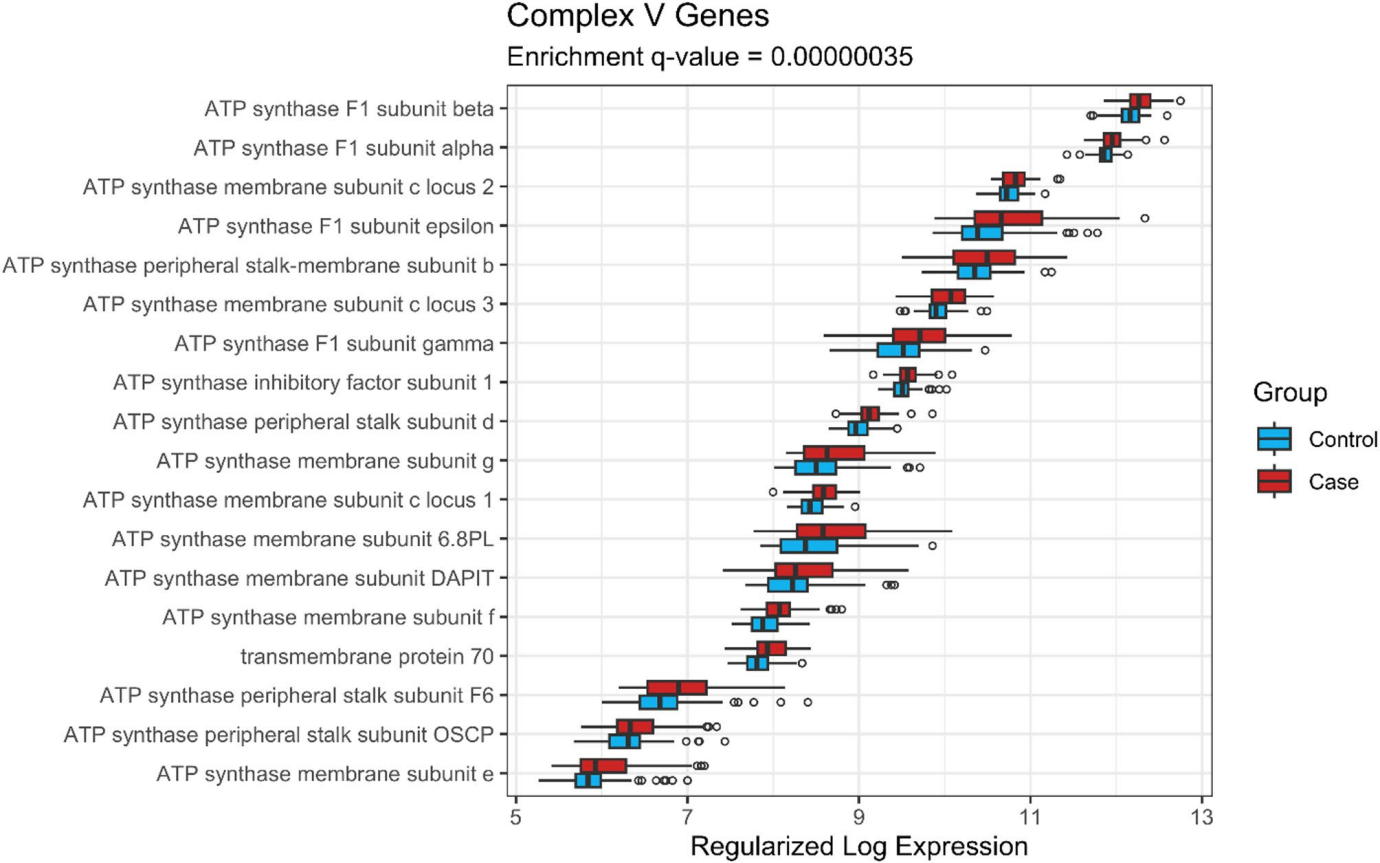
Expression of genes of Complex V that were in the leading edge of the gene set enrichment analysis

Expression by case-control status of genes of Complex V that were in the leading edge (LE) of the GSEA are shown in Fig. 4, and those of LE genes of Complexes I, III, and IV are shown in Supplementary Figs. 3–5, respectively. As can be observed in Fig. 4, the model-adjusted average expression of the 28 ATP synthase subunit (Complex V) LE genes was higher in cases than controls (ranging from 1.12- to 1.58-fold higher). Likewise, the expression of the 27 NADH: ubiquinone oxidoreductase subunit (Complex I) LE genes was higher in cases than controls (ranging from 1.09- to 1.68-fold higher—see Supplementary Fig. 3). Similar observations can be made for the LE genes of the ubiquinol-cytochrome c oxidoreductase complex (Complex III, Supplementary Fig. 4) and the cytochrome c oxidase subunits (Complex VI, Supplementary Fig. 5).

### Secondary analysis of BAX/BCL-2 gene expression ratio at the mRNA-level

**Fig. 5:**
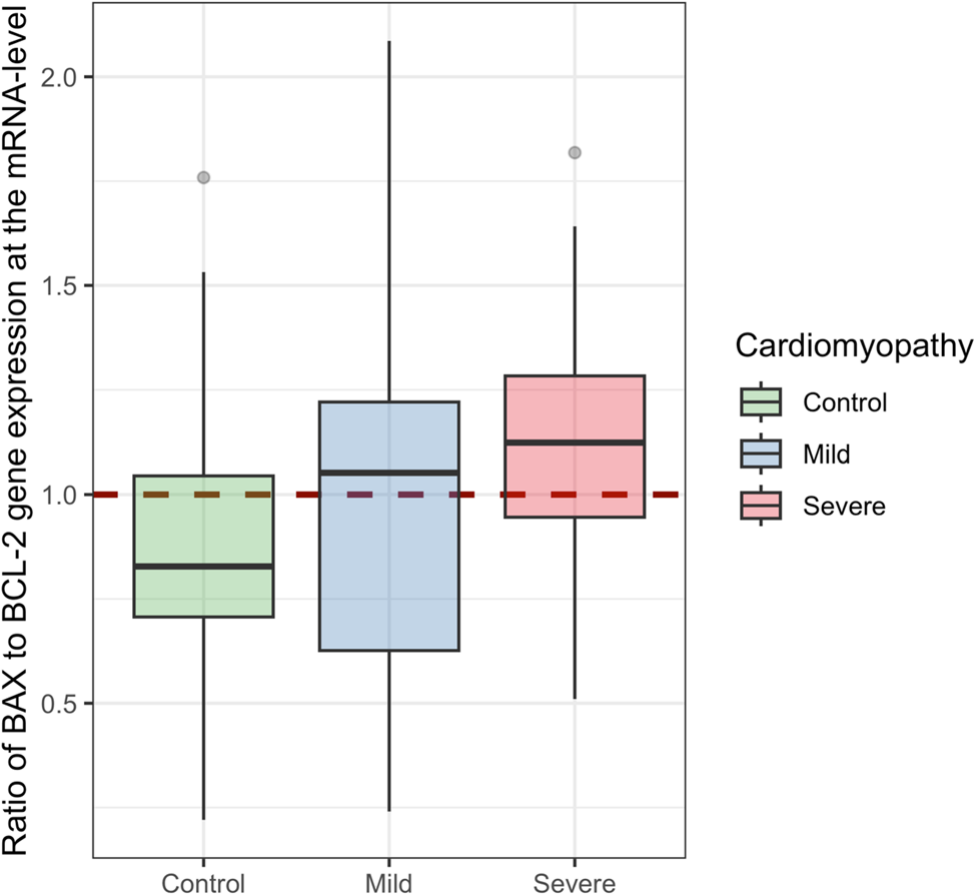
Boxplot of the ratio of *BAX* to *BCL-2* gene expression at the mRNA-level by group

In our secondary analysis of the ratio of *BAX* to *BCL-2* gene expression at the mRNA-level, cases were found to have 1.21-fold higher ratio (95% CI, 1.04–1.39, *p*-value = 0.01). To evaluate whether higher level of this marker of apoptotic susceptibility was related to severity of cardiomyopathy, we regressed the ratio on a three-level indicator of participant status: control, mild cardiomyopathy, and severe cardiomyopathy. We observed that the ratio of *BAX* to *BCL-2* gene expression increased linearly (*p*-value = 0.009 for linear slope) and was 1.14-fold higher in cases with severe cardiomyopathy compared to mild cardiomyopathy (95% CI, 1.03–1.26). A boxplot of *BAX* to *BCL-2* ratio by group is shown in Fig. 5.

### Differential expression between severe and mild cardiomyopathy

In our secondary analysis comparing severe vs. mild cardiomyopathy, 470 genes (out of 16,913) were differentially expressed between severe cardiomyopathy cases and mild cardiomyopathy cases (Supplementary Table 1). Of the 1,043 genes that coded for mitochondrial proteins, 40 were differentially expressed. In terms of agreement between these results and the primary analysis comparing cases and controls, the correlation between test statistics was 0.84 for all genes (95% CI, 0.84–0.85) and 0.87 (95% CI, 0.86–0.89) for genes encoding mitochondrial proteins. This agreement over genes coding for mitochondrial proteins is shown in Supplementary Fig. 6.

### Exploratory analysis of Stress-Associated gene sets

We evaluated gene sets associated with integrated stress response (ISR), unfolded protein response (UPR), and heat shock response (HSR) to assess their enrichment in our data. The following biological process gene ontology sets were utilized in a gene set enrichment analysis: GO:0140467 integrated stress response signaling, GO: 0006986 response to unfolded protein, GO:0031072 heat shock protein binding. The un-adjusted GSEA *p*-values for these sets were 0.03, 0.0005, and 0.07, respectively.

## Discussion

Given the centrality of mitochondria to the pathophysiology of the failing heart, our identification of mitochondrial processes and genes coding for mitochondrial proteins that are associated with anthracycline-induced cardiomyopathy is important for understanding this late complication of anthracycline treatment in survivors of childhood cancers. This work complements our previous effort undertaken with the same study participants towards identifying gene expression signatures at the mRNA-level associated with the development of anthracycline-induced cardiomyopathy [21]. In that work we did not focus specifically on the role of mitochondria but rather identified all differentially expressed genes. Further, our collaborators conducted functional validation of a significant finding by the knockout of lactate dehydrogenase A. Specifically, using human-induced pluripotent stem cell-derived cardiomyocytes with CRISPR/Cas9 knockout of this gene it was demonstrated that the absence of lactate dehydrogenase A increased sensitivity to doxorubicin.

Impaired functioning of mitochondria is a hallmark of cardiomyopathy [31–34]. Consistent with this, we observed that twice as many genes coding for mitochondrial proteins were differentially expressed between cases and controls compared to genes not known to encode mitochondrial proteins. Our observation that 97% of these differentially expressed genes were upregulated (as opposed to downregulated) in cardiomyopathy is a remarkable finding. This finding of systematic upregulation at the mRNA level was specific to genes coding for mitochondrial proteins; the distribution of fold-changes for genes not coding for mitochondrial proteins was centered near zero (increased or decreased mRNA level between cases and controls was similarly likely). This provides evidence that upregulation of genes involved in mitochondrial processes is associated with the development of cardiomyopathy in survivors who were treated with anthracyclines. This may reflect a pattern of mitochondrial dysregulation in which impaired mitochondrial function triggers increased transcription of genes encoding mitochondrially localized proteins and mitochondrial biogenesis as a compensatory mechanism. Prior studies have demonstrated that such upregulation can occur in the setting of oxidative phosphorylation impairment and energetic stress, resulting in a dysfunctional mitochondrial phenotype rather than functional recovery [35]. We emphasize that inferences about mitochondrial state remain speculative without accompanying functional validation in the context of anthracycline-mediated cardiomyopathy. Interestingly, the upregulation of genes involved in mitochondrial processes appears limited to nuclear-encoded genes, as each of the 16 mitochondrial DNA-encoded genes evaluated had scant evidence for differential expression (all q-values >0.28).

We observed significant evidence for differences in ROS metabolism in those who developed anthracycline-induced cardiomyopathy compared to those who did not. Differences were observed in the expression of peroxiredoxin genes, thioredoxin genes, a superoxide reductase gene, and genes involved in glutathione metabolism. Peroxiredoxins catalyze the breakdown of hydrogen peroxide as part of the response to ROS. Following the oxidation of a peroxiredoxin in the reduction of hydrogen peroxide, a disulfide bond is formed with a neighboring peroxiredoxin [36]. To regenerate the peroxiredoxins, thioredoxin can reduce this disulfide bond, and then be regenerated by thioredoxin reductase using NADPH. Microsomal glutathione S-transferase 1 (MGST1) is also involved in the defense against ROS and is localized to the mitochondrial outer membrane and the endoplasmic reticulum [37]. Superoxide dismutases (SOD) are involved in scavenging superoxide radicals [38], and SOD1 is localized to multiple cellular locations including mitochondria [39]. Our observation that peroxiredoxin 2, 3, 4, and 5; thioredoxin 2; MGST1; and SOD1 were upregulated in cases suggests that responses to ROS are up-regulated in anthracycline-induced cardiomyopathy, long after acute treatment with anthracyclines.

Differences in expression of genes involved in iron transport, storage, metabolism, and heme synthesis were associated with anthracycline-induced cardiomyopathy. Ferredoxin reductase, an up-regulated gene in those with cardiomyopathy, catalyzes the transfer of electrons from NADPH to ferredoxin which then transfers electrons to cytochrome P450 [40, 41] or for use in iron-sulfur cluster biogenesis [42]. Knockdown of this gene has provided evidence that ferredoxin reductase is required for the synthesis of iron-sulfur clusters and reduced expression leads to mitochondrial iron overload [41]. Iron accumulation in mitochondria following anthracycline treatment has been recognized as a mediator of cardiotoxicity [5]. Further, work by Ichikawa, et al. noted that mitochondria isolated from patients with anthracycline-induced cardiomyopathy have higher levels of iron than hearts from patients with cardiomyopathy not associated with anthracycline treatment [5]. Additionally, the protein Fhit interacts with ferredoxin reductase and is hypothesized to directly block the ferredoxin reductase electron transfer that modulates ROS levels as well as ROS-induced apoptosis [43]. Upregulation of ferredoxin reductase in cases with anthracycline-induced cardiomyopathy in our study may thus be a response to iron accumulation that occurs with anthracycline treatment.

Heme biosynthesis genes were observed to be upregulated in those who developed cardiomyopathy. Aminolevulinic acid synthase 2 (encoded by the *ALAS2* gene), which catalyzes the first and rate limiting step of heme biosynthesis [44], and ferrochelatase, which catalyzes the last step, were both significantly up-regulated. A recent study demonstrated that acute treatment of cardiomyocytes with doxorubicin downregulates *ALAS2*, while simultaneously upregulating *ALAS1* in a dose-dependent manner [45]. Additionally, in work by Khechaduri, et al. [46], heme levels were observed to be elevated in failing human hearts from patients undergoing heart transplant. In those failing hearts *ALAS2* gene expression and protein levels were elevated compared to control hearts without heart failure. Finally, Khechaduri, et al. [46], utilized a cardiac myoblast model to evaluate the relationship between overexpression of *ALAS2*, heme, ROS, and apoptosis. They noted that overexpression resulted in higher levels of heme, increased generation of ROS, and an increase in apoptosis. Our findings of upregulation of key enzymes in the heme biosynthesis may thus portend higher levels of ROS and apoptosis in anthracycline-induced cardiomyopathy.

Predominately increased expression of genes encoding mitochondrial mediators of apoptosis in those who develop cardiomyopathy is a significant finding of the current work. The Bcl-2 protein family is central to the intrinsic pathway of apoptosis [47] and is characterized by the presence of Bcl-2 homology domains [48]. Two of the family members, BAX and BAK form pores in the mitochondrial outer membrane that ultimately result in the transport of cytochrome *c* into the cytoplasm [49], a critical step leading to cell death. We note that the gene coding for BAX was upregulated and is in the LE of the annotated process. We evaluated the ratio of *BAX* to *BCL-2* gene expression at the mRNA-level given that this ratio has been hypothesized to demarcate the induction of apoptosis and has been utilized in previous studies as a marker of apoptotic susceptibility [50, 51]. Our observation that on average this ratio is highest in severe cardiomyopathy, lowest in controls without cardiomyopathy, and intermediate in mild cardiomyopathy suggests that treatment with anthracyclines may tip the balance towards continuing apoptosis in anthracycline-induced cardiomyopathy. *BIK*, another family member that was upregulated and in the LE, has been shown to induce apoptosis through the recruitment of DRP1, which results in cytochrome *c* release from mitochondria [52, 53]. In contrast to *BAX* and *BIK*, *BOK* was downregulated. BOK shares multiple domains present in BAX and BAK and significant amino sequence similarity with those family members [54]. However, BOK does not appear to mediate apoptosis in response to the same apoptotic stimuli as BAX and BAK [55]. Instead there is evidence that BOK is involved in the unfolded protein response [56], and the induction of apoptosis in response to endoplasmic reticulum stress [57].

In recognition of the role that mitochondrial stress responses may play in anthracycline-induced cardiomyopathy, we explored enrichment of gene sets involved in integrated stress response (ISR), unfolded protein response (UPR), and heat shock response (HSR). We observed modest evidence of enrichment for these pathways, with the most significant signal observed for genes involved in the unfolded protein response followed by ISR and HSR.

Interferon Alpha Inducible Protein 27 (IFI27, synonym ISG12a) was observed to be upregulated 6-fold in cardiomyopathy cases versus controls. IFI27 is inducible and highly expressed following type I interferon (specifically IFN-β) treatment, although IFN-γ increases expression to a lower degree [58]. IFI27 increases apoptosis via the intrinsic pathway following DNA-damage, an effect that is reduced when expressed with Bcl-2 [58]. The apoptotic response mediated by IFI27 follows cell cycle arrest, is caspase dependent and p53 independent [59]. Recently IFI27 has been shown to inhibit Wnt/β-catenin signaling which suppresses PD-L1 expression [60]. Taken together, these findings suggest that cases with anthracycline-induced cardiomyopathy may be experiencing an ongoing maladaptive immune response. HtrA2 (also known as Omi) is a serine protease and chaperone that in mitochondria inhibits the accumulation of unfolded proteins and may trigger apoptosis [61–63] and was observed to be upregulated in cases with cardiomyopathy. In a transgenic mouse model devised by Wang et al. [63], overexpression of cardiac-specific HtrA2 increased myocardial apoptosis. Further, in that study, overexpression resulted in salient phenotypic changes including both systolic and diastolic dysfunction.

Defects in OXPHOS / electron transport chain components in cardiomyopathy have long been documented [31–34]. In the current work we observed upregulation of genes coding for proteins of ETC Complexes I, III, IV, and V. As reviewed by Sheeran and Pepe [64], the activity of Complexes I, III, and IV along with some TCA cycle enzymes are decreased in heart failure. In their analysis of explanted hearts from patients with heart failure, they observed an increase in oxidative modification of proteins, especially redox centers, of ETC complexes [65]. Assuming that increased ROS results in oxidative damage to proteins of the ETC, increased expression of the same proteins may represent a compensatory mechanism in response to diminished energy production.

Some of the findings described in the current work have been observed in gene expression and transcriptomics studies of heart failure of other etiologies not associated with anthracycline exposure. Sweet, et al. conducted a transcriptome analysis comparing dilated cardiomyopathy and ischemic heart failure to non-failing hearts [66]. In their pathway analysis using IPA’s (Ingenuity Pathway Analysis) Canonical Pathways, “Mitochondrial Dysfunction” and “Oxidative Phosphorylation” emerged as the top two pathways with greatest evidence of enrichment. Hahn, et al. compared gene expression between cases with heart failure with preserved ejection fraction (HFpEF) and donors without heart failure. In their Gene Ontology Biological Process-based enrichment analysis, the majority of up-regulated processes were mitochondrial localized, for example, “NADH dehydrogenase complex assembly” [67].

### Limitations

A significant limitation of the current work is the source of biological samples. Inferences about the role of mitochondria in cardiomyopathy would be most relevant if made using cardiomyocytes, however such sampling from human participants would be highly invasive. The relationship between cardiomyocyte gene expression and mRNA expression levels quantified from whole blood is not available in the current work. The RNA isolation procedure used in this study does not account for the cellular composition of the whole blood samples. As a result, the findings may also reflect differences in cell composition between survivors who developed cardiomyopathy and those who did not. While we have adjusted the models underlying the differential expression analyses for some exposures (e.g., anthracycline dose and chest radiation), the risk of residual confounding from omitted variables related to mitochondrial biology remains. Additionally, as with all studies of gene expression at the mRNA-level, the target of interest is indirectly studied. Specifically, we are interested in mitochondrial function, yet multiple layers of regulation (e.g., protein translation and post-translational modification) exist between mRNA and functional proteins. The current study does not include proteomic or functional assays to validate mRNA-level findings. As such, conclusions regarding mitochondrial activity or dysfunction remain speculative and require follow-up experimental confirmation. Our analysis of apoptotic susceptibility relies on mRNA expression of selected apoptotic regulators and does not capture broader apoptotic pathway activity or specificity; a more comprehensive or functional analysis will be required to validate and contextualize these findings. Finally, the findings of this work have not yet been independently replicated, especially in the context of anthracycline-induced cardiomyopathy, and should therefore be interpreted with appropriate caution.

## Conclusions

In this work we identified 12 mitochondrial processes that are predominately up-regulated in peripheral blood in cancer survivors with anthracycline-induced cardiomyopathy. These included complexes of the electron transport chain, heme synthesis, metabolism of reactive oxygen species, apoptosis, mitophagy, and autophagy. Additionally, we observed that a measure of pro-apoptotic balance (BAX to BCL-2) was highest in severe cardiomyopathy, intermediate in mild cardiomyopathy, and lowest in survivors who did not develop cardiomyopathy.

## Supplementary Information

Below is the link to the electronic supplementary material.

Supplementary Material 1

## Data Availability

The data discussed in this article have been deposited in the NCBI Gene Expression Omnibus and are accessible through GEO Series accession number GSE218276 (https://www.ncbi.nih.gov/geo/query/acc.cgi?acc=GSE218276).

## Abbreviations

DEG: Differentially Expressed Gene
DEG-M: Differentially Expressed Gene coding for a Mitochondrial protein
DEG-NM: Differentially Expressed Gene Not known to code for a Mitochondrial protein
DNA: Deoxyribonucleic acid
EDTA: Ethylenediaminetetraacetic acid
GSEA: Gene Set Enrichment Analysis
LE: Leading Edge
mRNA: Messenger RNA
OXPHOS: Oxidative Phosphorylation
ROS: Reactive Oxygen Species
RNA: Ribonucleic acid
RNA-Seq: RNA Sequencing
