# Supplementary material for: Anthracycline-induced cardiomyopathy in childhood cancer survivors is associated with gene signatures of mitochondrial dysfunction—a COG ALTE03N1 report
Source: Cardiooncology. 2025 Oct 6;11:87. doi: 10.1186/s40959-025-00391-w (PMC12502420; doi:10.1186/s40959-025-00391-w)

**Title:** Anthracycline-Induced Cardiomyopathy in Childhood Cancer Survivors is Associated with Gene Signatures of Mitochondrial Dysfunction—A COG ALTE03N1 Report

**Authors:** Patrick J. Trainor, PhD, MS, MA^1,2^, Purnima Singh, PhD, MS, MSPH^1,3^, Xuexia Wang, PhD^4^, Noha Sharafeldin, PhD^1^, Liting Zhou, MS^1^, Lindsey Hageman, MPH^1^, Saro H. Armenian, DO, MPH^5^, Jill P. Ginsberg, MD^6^, Douglas S. Hawkins, MD^7^, Frank G. Keller, MD^8^, Melissa M. Hudson, MD^9^, Joseph P. Neglia, MD^10^, Wendy Landier^1,3^, PhD, Smita Bhatia, MD, MPH^1,3^

**Supplementary Table 1:** Results of the differential expression analysis. 16,913 genes were included in the analysis. Click [here](https://bioinfoscientistcom.sharepoint.com/:x:/s/BioinfoScientistExternal/EWQ9KTdUI1pAh_wF_qfClwYBe731rkD1-8XMDZYg8-f4kA?e=Cn0Z3o) for file.

**Supplementary Table 2:** 196 genes were in the leading edge of the running score for one or more significantly enriched mitochondrial processes. Click [here](https://bioinfoscientistcom.sharepoint.com/:x:/s/BioinfoScientistExternal/EQSu2IAZLSVIvoovpMw9XuMBFHWJbXokzHVvTGK4ZR9pKQ?e=EWcAl3) for file.

**Supplementary Figure 1:** Running enrichment score plots for top two processes (Mitochondrial Ribosome and Complex V).


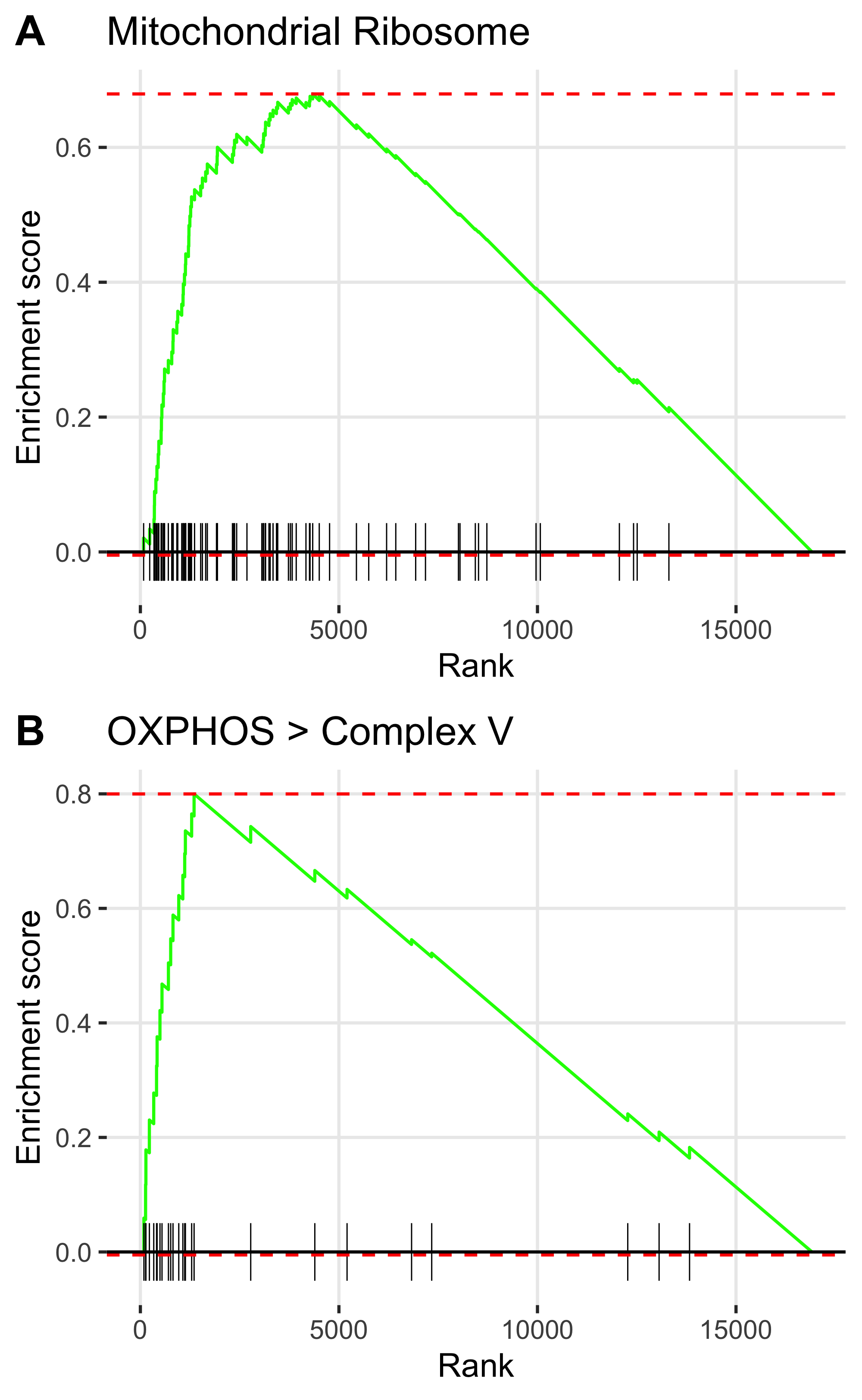


**
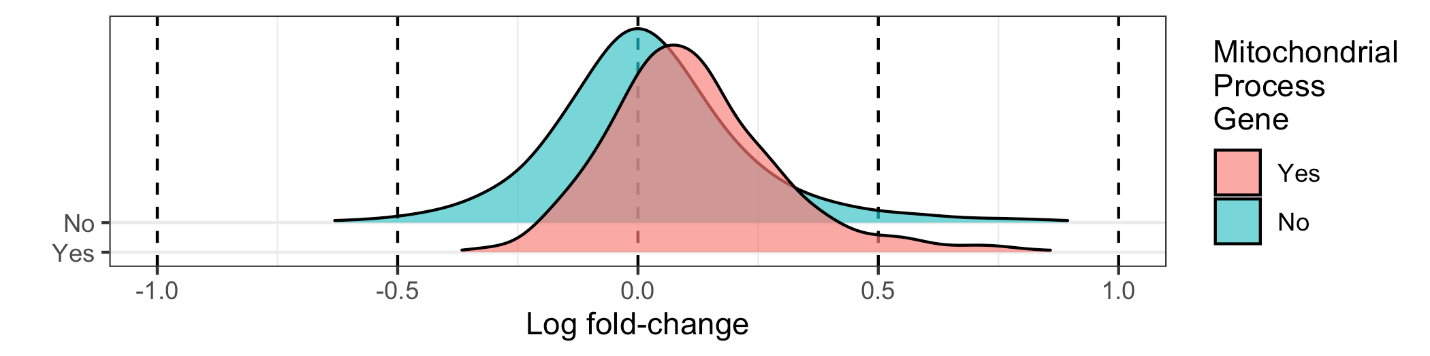
Supplementary Figure 2:** Distribution of log fold-changes for the 16,913 genes colored by whether each gene is involved in a mitochondrial process or not.

**Supplementary Figure 3:** Expression of genes of Complex I that were in the leading edge of the gene set enrichment analysis.

**
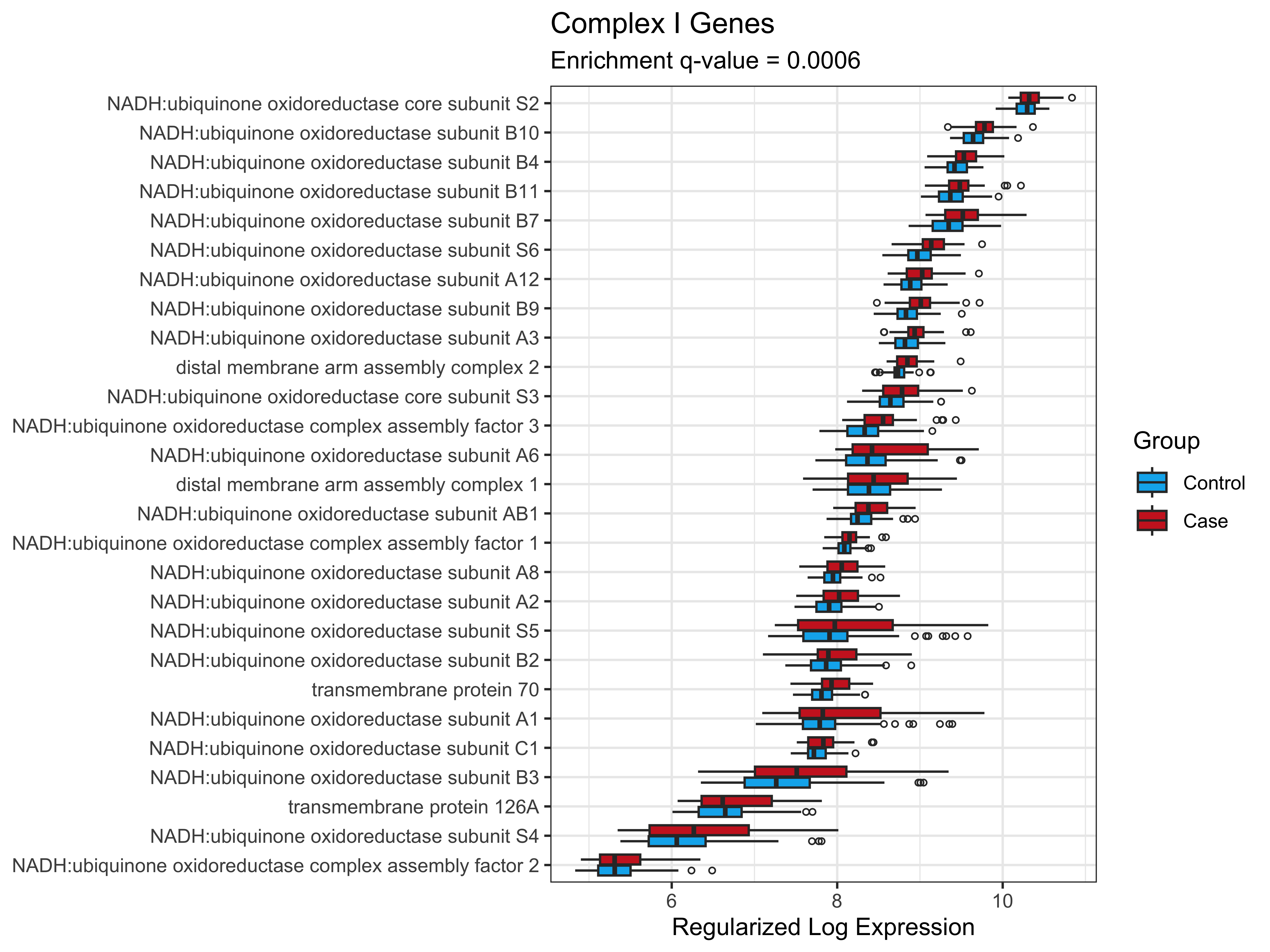
**

**Supplementary Figure 4:** Expression of genes of Complex III that were in the leading edge of the gene set enrichment analysis.

**
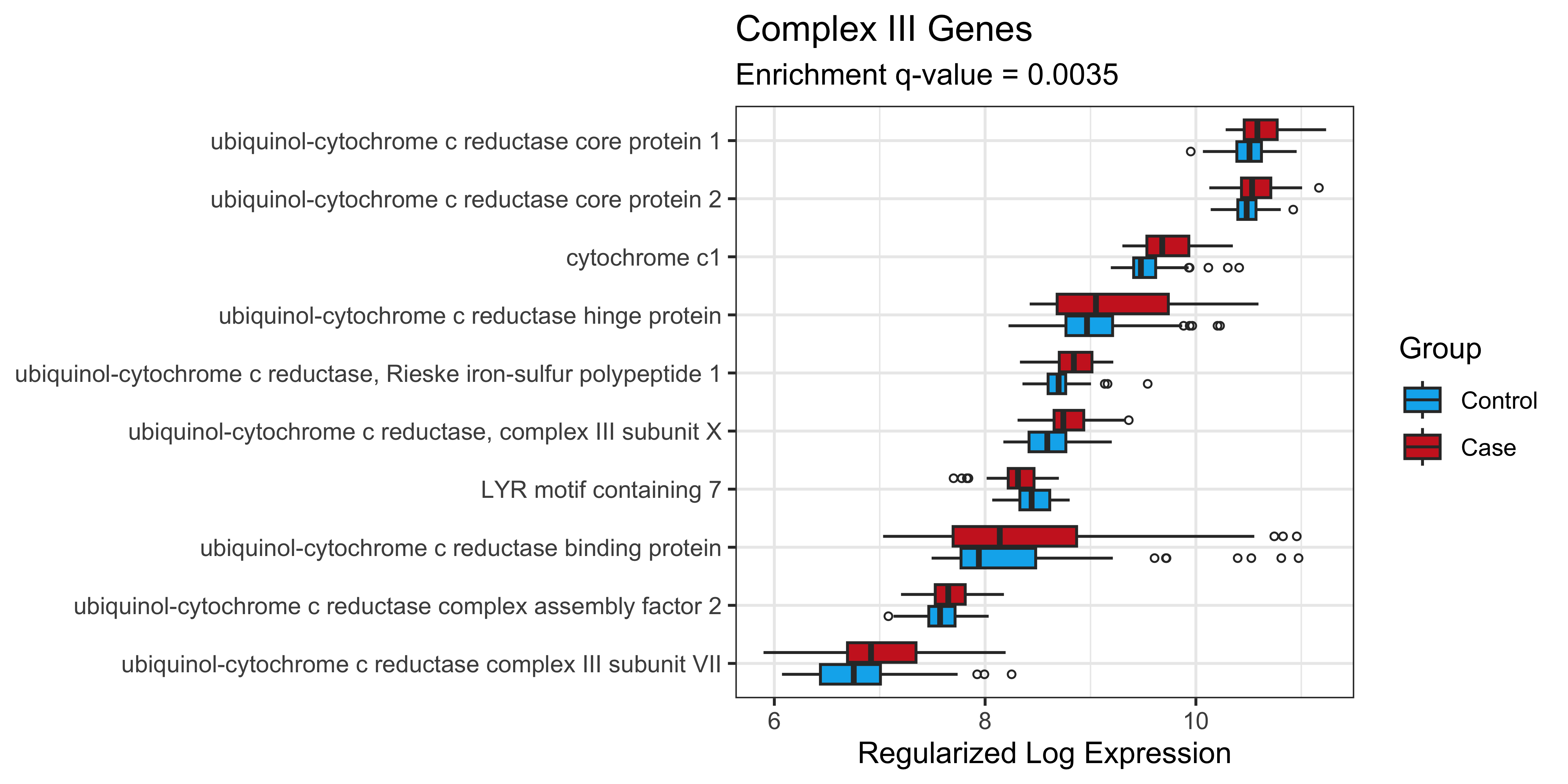
**

**Supplementary Figure 5:** Expression of genes of Complex IV that were in the leading edge of the gene set enrichment analysis.

**
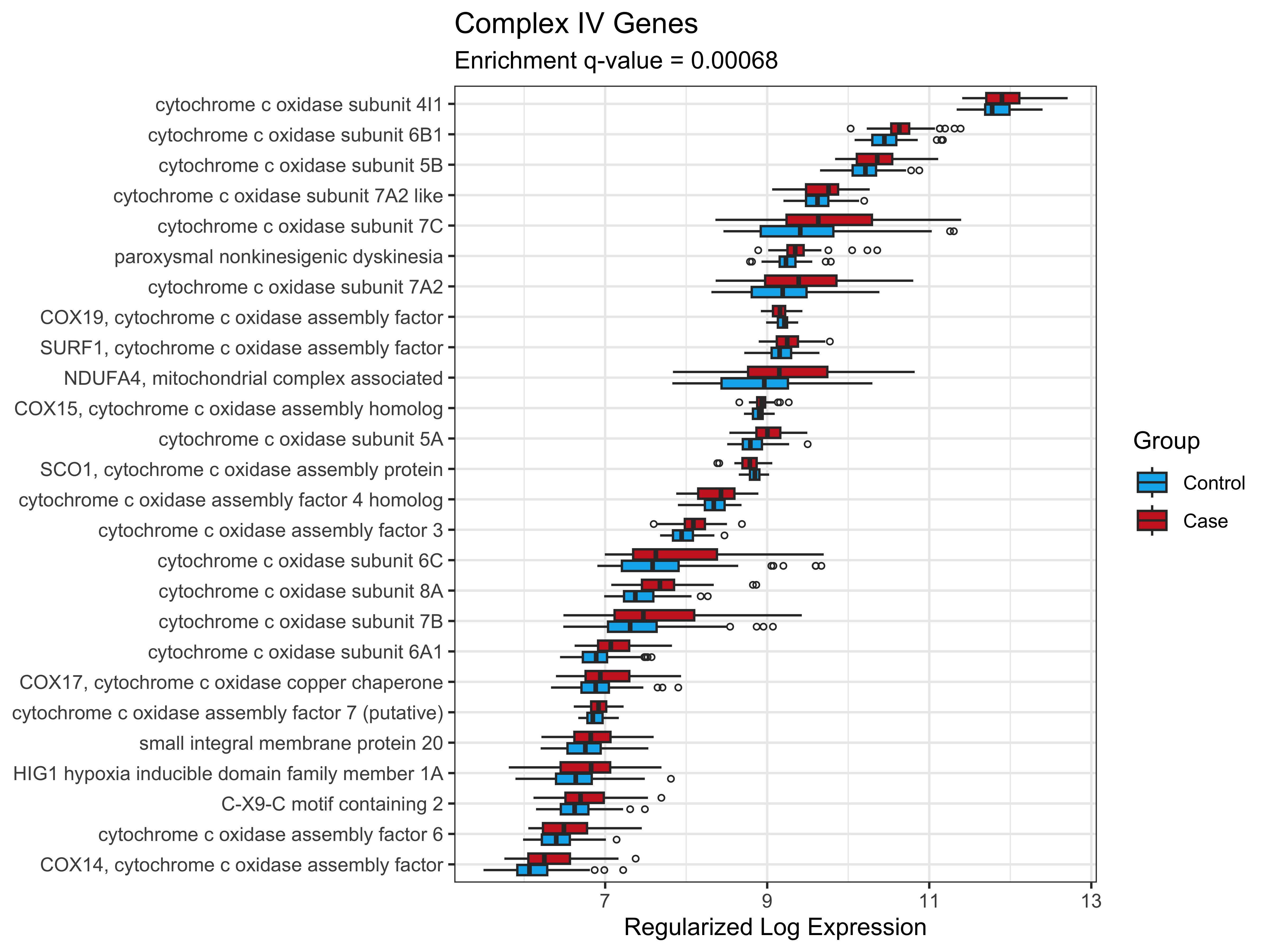
**

**Supplementary Figure 6:** Agreement between test statistics for genes coding for mitochondrial proteins in the primary and secondary analyses. The primary analysis compared cases with cardiomyopathy and controls. The secondary analysis compared severe versus mild cardiomyopathy.


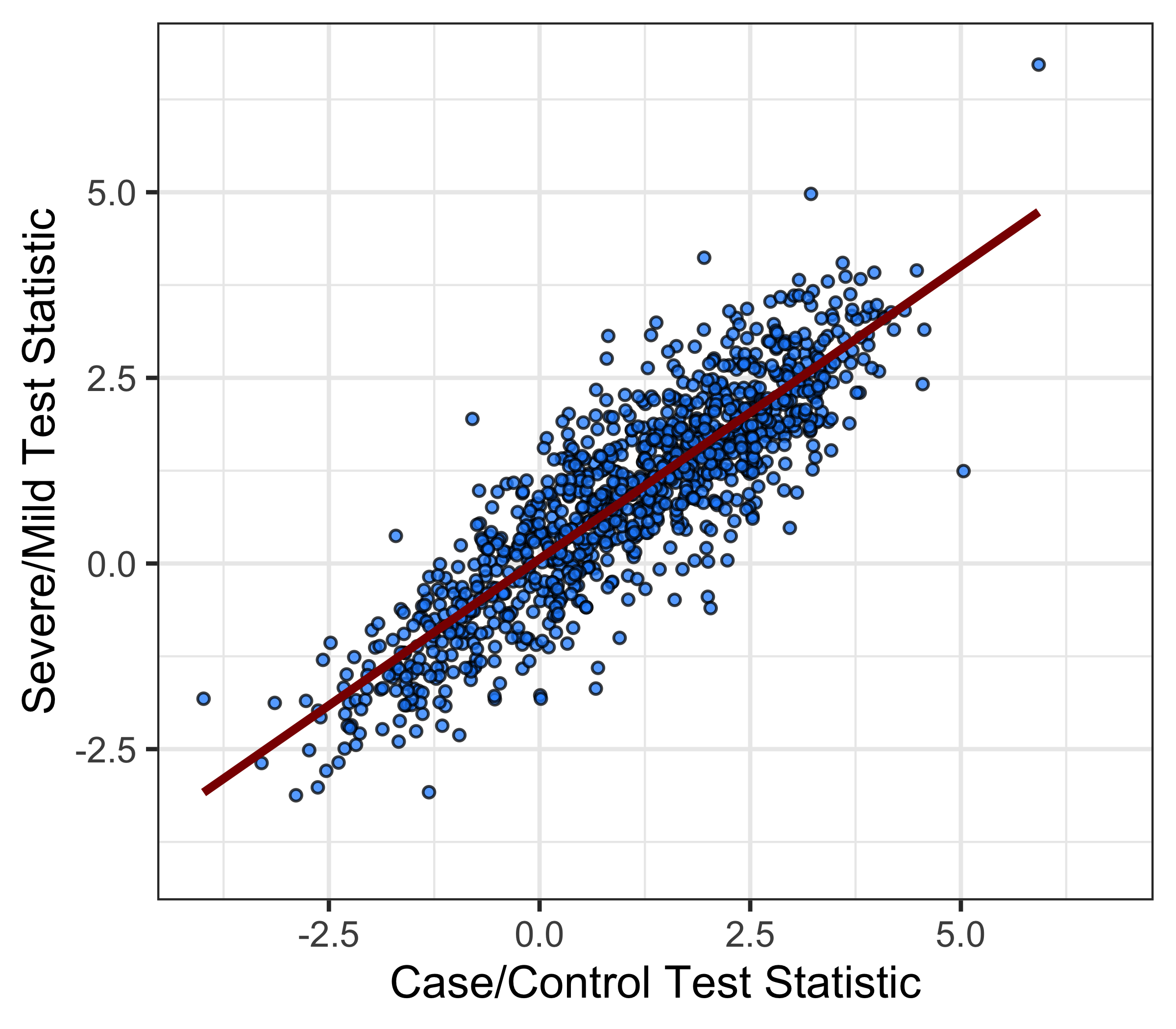

Supplement: Supplementary file 1 — Supplementary Material 1 [file 40959_2025_391_MOESM1_ESM.docx]
